# The Contribution of Former Work-Related Activity Levels to Predict Physical Activity and Sedentary Time during Early Retirement: Moderating Role of Educational Level and Physical Functioning

**DOI:** 10.1371/journal.pone.0122522

**Published:** 2015-03-31

**Authors:** Delfien Van Dyck, Greet Cardon, Benedicte Deforche, Ilse De Bourdeaudhuij

**Affiliations:** 1 Research Foundation Flanders, Brussels, Belgium; 2 Ghent University, Department of Movement and Sports Sciences, Ghent, Belgium; 3 Ghent University, Department of Public Health, Ghent, Belgium; Institute of Tropical Medicine (NEKKEN), Nagasaki University, JAPAN

## Abstract

**Background:**

The transition to retirement introduces a decline in total physical activity and an increase in TV viewing time. Nonetheless, as more time becomes available, early retirement is an ideal stage to implement health interventions. Therefore, knowledge on specific determinants of physical activity and sedentary time is needed. Former work-related physical activity has been proposed as a potential determinant, but concrete evidence is lacking. The aim of this study was to examine if former work-related sitting, standing, walking or vigorous activities predict physical activity and sedentary time during early retirement. Additionally, moderating effects of educational level and physical functioning were examined.

**Methods:**

In total, 392 recently retired Belgian adults (>6 months, <5 years) completed the International Physical Activity Questionnaire, the SF-36 Health Survey and a questionnaire on sociodemographics and former work-related activities. Generalized linear regression analyses were conducted in R. Moderating effects were examined by adding cross-products to the models.

**Results:**

More former work-related sitting was predictive of more screen time during retirement. Lower levels of former work-related vigorous activities and higher levels of former work-related walking were associated with respectively more cycling for transport and more walking for transport during retirement. None of the predictors significantly explained passive transportation, cycling and walking for recreation, and leisure-time moderate-to-vigorous physical activity during retirement. Several moderating effects were found, but the direction of the interactions was not univocal.

**Conclusions:**

Former-work related behaviors are of limited importance to explain physical activity during early retirement, so future studies should focus on other individual, social and environmental determinants. Nonetheless, adults who previously had a sedentary job had higher levels of screen time during retirement, so this is an important subgroup to focus on during interventions. Because of the inconsistent moderating effects of educational level and physical functioning, no clear recommendations can be formulated.

## Introduction

In developed countries, life expectancy has increased steadily over the last decades [1]. As the prevalence of major chronic diseases increases with age, the increased life expectancy induces a large medical burden and a rise in health care costs [2]. To prevent the development of chronic diseases (e.g. sarcopenia) in older adults, a healthy lifestyle with sufficient physical activity and limited sedentary time is needed [3–5]. Nevertheless, physical activity typically declines with increasing age [6], while sedentary time (e.g. TV viewing, reading) increases [7].

The transition to retirement is an important stage in an adult’s life. In Belgium, the formal retirement age varies between 58 and 65 years (dependent on the type of occupation). Research has shown that retirement introduces a decline in total physical activity, probably caused by a decrease in work- and transport-related physical activity, that is not sufficiently compensated by an increase in leisure-time physical activity [8–10]. Little is known about the change of sedentary time during early retirement: some previous studies reported that retirement was associated with an increase in TV viewing time [10–12], but the impact on other sedentary behaviors remains unclear. Nonetheless, retirement offers opportunities to develop healthy lifestyles, as the time previously spent working can be spent on other activities and new habits can be developed [13]. Furthermore, persons who are about to retire or retired recently seem to be particularly receptive to behavior change [14]. Consequently, early retirement is an ideal stage to implement interventions to prevent older adults from lapsing into an inactive and/or sedentary lifestyle.

Before interventions can be developed and implemented, it is important to identify the determinants of physical activity and sedentary time during early retirement. However, the current evidence base is very limited. Results of qualitative research conducted through focus groups, showed that individual-level factors (e.g. social norm and beliefs on ageing and retirement, need for personal challenges, perceived health benefits of physical activity, financial constraints) and interpersonal factors (e.g. social interactions, social support) can be important motives for (insufficient) physical activity in recently retired adults [15,16].

Furthermore, it has been suggested that former work-related physical activity can be an important predictor of physical activity levels during retirement [15], but concrete evidence is lacking. Some previous studies showed that physical activity levels of manual workers (who are supposed to be more active at work than non-manual workers) decreased more strongly after retirement compared with physical activity levels of non-manual workers [9,17]. Nonetheless, none of these studies actually examined the amount of former occupational physical activity, so it remains unclear if former occupational physical activity truly is a determinant of physical activity during retirement. It has been hypothesized that adults who had a physically demanding job attach low personal value to leisure-time physical activity because of an inherent belief that recreational physical activity is a waste of time [15]. Moreover, they may experience retirement as a period of well-deserved rest, after an active professional career.

In the context of future interventions it would be very useful to gain further insight in these associations; if the formulated hypotheses are confirmed in empirical studies, interventions could already be implemented at the end of the working career, with a specific focus to subgroups who are more susceptible to low physical activity levels and high levels of sedentary time during early retirement (e.g. those with high levels of vigorous physical activity at work).

Consequently, the first aim of this study was to examine if former work-related sitting, standing, walking or vigorous activities predict physical activity and sedentary time during early retirement. Secondly, we examined the moderating effects of educational level and physical functioning on these associations. Educational level, as a proxy for socio-economic status (SES) was included as a moderator because individuals of higher SES probably have more financial resources to support physical activities after retirement [18], which may affect the examined associations. Physical functioning was included as a moderator because functional limitations are a prominent problem in ageing people, that needs to be taken into account when physical activity and sedentary time are examined [19]. Very specific physical activity measures (transport-related walking and cycling, leisure-time walking and cycling, and leisure-time moderate-to-vigorous physical activity (MVPA) were examined as outcome measures, because previous research showed that determinants are very behavior-specific [20]. Furthermore, functional limitations are probably more important in the relationship with MVPA or cycling than with walking.

## Methods and Materials

The present study was conducted in Ghent (250,000 inhabitants, 156.18 km^2^ (60.3 miles^2^), 1601 inhabitants/km^2^), Flanders, Belgium. Data were collected in two waves, a first wave in December 2012 and a second wave in May 2013.

### Ethics Statement

The study protocol was approved by the ethics committee of the Ghent University Hospital. Written informed consent was obtained from all participants.

### Procedures and participants

Retirement can be defined as ‘a permanent and complete withdrawal from the labor force’. However, in this study, also adults who were still doing voluntary work after retirement from their main occupation were included. In Flanders, the formal retirement age varies between 58 and 65 years (http://www.onprvp.fgov.be), but official records with information on retirement status are not publicly available. Consequently, in order to recruit a sufficient number of recently retired adults (>6 months, <5 years of retirement), the Public Service of Ghent selected a random sample of 7500 58–65 year old adults from the municipal register. All these adults received an invitation letter with information on the study (2500 adults in December 2012, 5000 adults in May 2013). After two weeks, a reminder was sent. Only adults who had been retired for more than 6 months but less than 5 years could participate in the study. Furthermore, as physical activity was the outcome variable in this study, participants had to be able to walk 100 meters without assistance in order to be eligible for the study. Adults who were willing to participate in the study and met the two inclusion criteria, were asked to confirm their participation by phone or email. In total, 428 eligible adults confirmed participation. Because it is unknown how many of the 7500 addressed adults were truly eligible to participate in the study, it is not possible to calculate a reliable response rate.

These adults received a postal questionnaire (with a pre-stamped envelope to return the questionnaire) including questions on socio-demographic characteristics, physical activity and sedentary time during retirement, physical and mental health, and former work-related physical activities. In total, 392 complete questionnaires (135 in December and 257 in May) were returned.

### Measures

#### Physical activity and sedentary time

Self-reported physical activity was measured with the International Physical Activity Questionnaire (IPAQ; long past seven days version). Physical activity assessed by the IPAQ showed good reliability (intra-class correlations range from 0.46 to 0.96) and fair-to-moderate criterion validity compared against accelerometers (median ρ 0.30) in a 12-country study [21]. Frequency (number of days) and duration (minutes/day) of physical activity in different domains were queried. Based on this information, separate estimates of weekly minutes of cycling for transport, walking for transport, cycling for recreation, walking for recreation and leisure-time MVPA were calculated. Self-reported minutes/week of screen time and passive transport were assessed using a translated (Flemish) version of the leisure-time sedentary behavior questionnaire developed by Salmon and colleagues [22]. The English-language version of the questionnaire has fair to excellent reliability (intra-class range from 0.56 to 0.82). Concurrent validity, assessed against a three-day behavioral log was fair-to-moderate, with rho’s ranging from 0.20 to 0.60 [22].

#### Former work-related behaviors

To obtain information on former work-related sitting, standing, walking and vigorous physical activity, the work-related questions of the usual week version of the long IPAQ were slightly modified. In the original IPAQ, minutes/week of work-related walking and vigorous physical activity are assessed. For the purpose of the present study min/week of former work-related sitting and standing were added. All questions concerned the participants’ last job before they retired.

#### Socio-demographic information and physical functioning

Self-reported socio-demographics included sex, age, weight, height, current marital status (alone, married, living together, widowed, divorced), and educational level (primary, secondary, tertiary education). BMI was calculated by dividing the weight (kg) by the height (m) squared. For the moderation analyses, educational level was dichotomized into having a college or university degree versus having no college or university degree. Marital status was dichotomized into living with or without a partner. Self-reported physical functioning was assessed with the physical functioning subscale of the Short Form 36 item Survey (SF-36) [23]. This subscale comprises ten activities (e.g. moderate activities, climb several flights, bend or kneel) and participants were asked to report on a 3-point scale whether or not they were restricted by their physical health to perform these activities (severely limited; somewhat limited; not limited). Subsequently, activities in which participants reported to be severely or somewhat limited were summed. For the moderation analyses, this measure was dichotomized at its median value (2 limitations), yielding the categories “functionally limited” (≥2 limitations) and “not functionally limited” (0–1 limitation).

### Statistical analyses

To study the main associations of former work-related activities with physical activity and sedentary time during retirement, two types of generalized linear regression models were used. First, because screen time and passive transport (dependent variables) were continuous variables with a normal distribution and almost no zero values, Gaussian models were used to model the associations with former work-related activities. Akaike’s Information Criterion (AIC) tests confirmed that Gaussian models were the best models to fit these data. From these regression models, beta-coefficients and 95% confidence intervals were reported. Second, when cycling/walking for transport/recreation and leisure-time MVPA were the dependent variables, zero-inflated negative binomial (ZINB) regression models with robust standard errors were used [24]. This was done because the dependent variables were positively skewed and contained an excess number of zero counts. Vuong tests supported the need to use zero-inflated regression models [25]. ZINB models evaluate the correlates of the odds of non-participation in walking/cycling for transport/recreation and leisure-time MVPA (logit model). Simultaneously, among those who did perform the respective physical activities, ZINB models evaluate the correlates of weekly minutes of the dependent variables. Hence, one ZINB model yields two regression coefficients for each independent variable: an odds ratio (OR) for the logit model and a negative-binomial regression coefficient, representing the proportional changes in min/week of physical activity with a one-unit increase in the independent variable for those participants engaging in the respective physical activities. Again 95% confidence intervals were reported.

More concretely, separate regression models were constructed for each dependent variable. In each regression model, sex, BMI and marital status were included as socio-demographic covariates. Furthermore, educational level and physical functioning (moderators) were included as independent variables, as well as minutes/day of former-work related sitting, standing, walking and vigorous physical activity.

To examine the moderating effects of educational level and physical functioning (dichotomous variables), the cross-products “*moderator x former work-related behavior”* were added to the regression models. A separate regression model was constructed for each moderating effect and next to the cross-products, the socio-demographic covariates, the respective moderator and former work-related behavior (e.g. sitting) were included in the model. In case of significant moderating effects (90% confidence intervals were used to determine significance because analyses with interaction terms have less power) [26], separated regression models were run to interpret the direction of the interactions (95% confidence intervals to determine significance). All analyses were conducted in R (R Development Core Team, 2013), using the packages “mass” [27], and “pscl” [28].

## Results

### Sample characteristics

Descriptive characteristics of the sample are presented in Table 1. Of the total sample, 50.9% was male, 73.5% lived with a partner, 54.0% had a college or university degree and 57.7% had two or more physical limitations. Mean age of the participants was 62.9 (standard deviation [SD] = 2.0) years, and mean BMI was 26.0 (SD = 4.6). Descriptive information on former work-related behaviors, sedentary time and physical activity during retirement is also shown in Table 1. Remarkably, many participants reported not to engage in any walking for recreation (40.3%), cycling for transport (55.9%), leisure-time MVPA (61.0%) or cycling for recreation (66.1%) during retirement.

**Table 1:** Socio-demographic sample characteristics, former work related behaviors, sedentary time and physical activity.

Variable	Total sample (n = 392)
Socio-demographic factors	
**Sex** (% male)	50.9
**Age** (mean [SD])	62.9 (2.0)
**BMI** (mean [SD])	26.0 (4.6)
**Marital status** (% with partner)	73.5
**Educational level** (% with college/university degree)	54.0
**Physical functioning**	
0–1 physical limitation	42.3
≥2 physical limitations	57.7
Former work-related behaviors (mean [SD])	
**Work-related sitting** (min/day)	182.1 (111.1)
**Work-related standing** (min/day)	139.6 (109.6)
**Work-related walking** (min/day)	64.0 (78.9)
**Work-related vigorous PA** (min/day)	60.8 (126.2)
Sedentary time during retirement (mean [SD])	
**Screen time** (min/week)	1432.5 (800.8)
**Passive transport** (min/week)	428.0 (491.0)
Physical activity during retirement (mean [SD])	
**Cycling for transport**	
Non-zero duration (min/week)^1^	158.4 (152.3)
% reporting no cycling	55.9
**Walking for transport**	
Non-zero duration (min/week)^1^	239.6 (229.3)
% reporting no walking	25.5
**Cycling for recreation**	
Non-zero duration (min/week)^1^	257.2 (236.8)
% reporting no cycling	66.1
**Walking for recreation**	
on-zero duration (min/week)^1^	257.5 (239.5)
% reporting no walking	40.3
**Leisure-time MVPA**	
Non-zero duration (min/week)^1^	263.5 (305.3)
% reporting no MVPA	61.0

MVPA: moderate-to-vigorous physical activity; PA: physical activity; SD: standard deviation

^1^ duration among those who did the behavior

### Main associations of socio-demographic covariates and moderators with sedentary behaviors and physical activity

The generalized linear Gaussian models (Table 2) showed that screen time was higher in participants with a higher BMI and participants having no college/university degree than in those with a lower BMI and having a college/university degree. Furthermore, male participants reported more passive transport than their female counterparts.

**Table 2:** Main associations of former work-related sitting, standing, walking and vigorous physical activity with screen time and passive transport during retirement (generalized linear Gaussian models).

Predictor	Screen time	Passive transport
	β (95%CI)	β (95%CI)
*Socio-demographic covariates*		
Sex (ref. male)	-146.30 (-25.80; 318.40)	**-114.00 (-225.01; -2.99)**
BMI	**20.66 (3.27; 38.05)**	2.30 (-8.70; 13.30)
Marital status (ref. without partner)	-0.55 (-186.75; 185.65)	-93.44 (-213.27; 17.57)
*Moderators*		
Educational level (ref. no college/univ degree)	**-184.30 (-352.13; -16.47)**	-104.41 (-213.05; 4.23)
Physical functioning (ref. <2 limitations)	99.31 (-63.55; 262.86)	-29.22 (-134.53; 76.09)
*Former work-related behaviors*		
Work-related sitting	**1.60 (0.58; 2.62)**	0.20 (-0.47; 0.87)
Work-related standing	-0.24 (-1.20; 0.72)	-0.05 (-0.68; 0.58)
Work-related walking	-0.03 (-1.17; 1.11)	-0.09 (-0.81; 0.64)
Work-related vigorous physical activity	0.67 (-0.15; 1.49)	-0.13 (-0.66; 0.40)

CI = confidence interval

The logit models showed that for cycling for transport, having a partner was associated with a 42% lower odds of being a non-participant (see Table 3). In other words, participants with a partner were more likely to cycle for transport. Similarly, having two or more physical limitations was associated with a 65% higher odds of being a non-participant, i.e. participants with two or more limitations were less likely to cycle for transport. For the other physical activity behaviors, the logit models can be interpreted similarly: Females were more likely to walk for transport than males, and participants with two or more physical limitations were less likely to participate in leisure-time MVPA than those with no or one physical limitation.

**Table 3:** Main associations of former work-related sitting, standing, walking and vigorous physical activity with physical activity during retirement (zero-inflated negative binomial models).

	Cycling for transport		Walking for transport	
	Logit model: OR of doing no cycling for transport^1^ e^b^ (95% CI)	Negative binomial model: min/week e^b^ (95% CI)	Logit model: OR of doing no walking for transport^2^ e^b^ (95% CI)	Negative binomial model: min/week e^b^ (95% CI)
*Socio-demographic covariates*				
Sex (ref. male)	0.99 (0.62; 1.58)	0.85 (0.65; 1.10)	**0.52 (0.30; 0.88)**	1.06 (0.84; 1.34)
BMI	1.04 (0.99; 1.09)	**0.98 (0.96; 0.99)**	1.02 (0.97; 1.08)	0.99 (0.96; 1.01)
Marital status (ref. without partner)	**0.58 (0.35; 0.98)**	**0.68 (0.49; 0.93)**	1.31 (0.71; 2.39)	0.91 (0.71; 1.17)
*Moderators*				
Educational level (ref. no college/univ degree)	0.89 (0.57; 1.41)	**0.75 (0.57; 0.98)**	1.09 (0.64; 1.84)	**0.78 (0.62; 0.99)**
Physical functioning (ref. <2 limitations)	**1.65 (1.06; 2.56)**	1.02 (0.79; 1.32)	1.17 (0.71; 1.93)	1.09 (0.87; 1.37)
*Former work-related behaviors*				
Work-related sitting	1.001 (0.999; 1.004)	0.998 (0.996; 1.000)	0.998 (0.995; 1.001)	0.999 (0.998; 1.000)
Work-related standing	0.999 (0.997; 1.002)	0.999 (0.998; 1.001)	0.999 (0.996; 1.002)	1.000 (0.999; 1.001)
Work-related walking	0.999 (0.996; 1.002)	1.000 (0.999; 1.002)	**0.996 (0.992; 0.999)**	**1.002 (1.001; 1.004)**
Work-related vigorous physical activity	1.000 (0.998; 1.002)	**0.998 (0.997; 0.999)**	1.001 (0.999; 1.003)	0.999 (0.998; 1.000)
	**Cycling for recreation**		**Walking for recreation**	
	Logit model: OR of doing no cycling for recreation^3^ e^b^ (95% CI)	Negative binomial model: min/week e^b^ (95% CI)	Logit model: OR of doing no walking for recreation^4^ e^b^ (95% CI)	Negative binomial model: min/week e^b^ (95% CI)
*Socio-demographic covariates*				
Sex (ref. male)	1.46 (0.90; 2.38)	**0.63 (0.47; 0.84)**	0.76 (0.48; 1.21)	0.85 (0.67; 1.08)
BMI	1.01 (0.96; 1.06)	0.99 (0.96; 1.01)	1.02 (0.98; 1.07)	0.97 (0.94; 0.99)
Marital status (ref. without partner)	0.58 (0.33; 1.00)	1.05 (0.72; 1.53)	0.84 (0.51; 1.38)	**0.69 (0.53; 0.90)**
*Moderators*				
Educational level (ref. no college/univ degree)	1.15 (0.72; 1.85)	1.02 (0.76; 1.37)	1.09 (0.86; 1.39)	1.09 (0.86; 1.39)
Physical functioning (ref. <2 limitations)	1.50 (0.95; 2.37)	1.13 (0.86; 1.50)	0.95 (0.61; 1.47)	0.86 (0.68; 1.08)
*Former work-related behaviors*				
Work-related sitting	1.000 (0.997; 1.003)	0.999 (0.997; 1.000)	0.999 (0.997; 1.002)	1.001 (0.999; 1.002)
Work-related standing	0.999 (0.996; 1.002)	0.999 (0.998; 1.001)	0.999 (0.997; 1.002)	1.000 (0.999; 1.001)
Work-related walking	1.001 (0.998; 1.005)	0.998 (0.996; 1.001)	0.998 (0.995; 1.001)	1.001 (0.999; 1.003)
Work-related vigorous physical activity	1.002 (0.999; 1.005)	0.999 (0.997; 1.001)	1.001 (0.999; 1.004)	1.000 (0.999; 1.002)
	**Leisure-time MVPA**			
	Logit model: OR of doing no leisure-time MVPA^5^ e^b^ (95% CI)	Negative binomial model: min/week e^b^ (95% CI)		
*Socio-demographic covariates*				
Sex (ref. male)	0.80 (0.15; 4.36)	**0.66 (0.48; 0.92)**		
BMI	1.00 (0.96; 1.05)	**0.98 (0.95; 0.99)**		
Marital status (ref. without partner)	1.29 (0.77; 2.14)	1.06 (0.75; 1.49)		
*Moderators*				
Educational level (ref. no college/univ degree)	0.63 (0.39; 1.01)	0.87 (0.62; 1.23)		
Physical functioning (ref. <2 limitations)	**2.39 (1.52; 3.76)**	0.79 (0.58; 1.09)		
*Former work-related behaviors*				
Work-related sitting	1.001 (0.998; 1.004)	1.000 (0.998; 1.002)		
Work-related standing	1.001 (0.998; 1.004)	1.000 (0.999; 1.002)		
Work-related walking	0.999 (0.996; 1.002)	1.001 (0.999; 1.003)		
Work-related vigorous physical activity	0.999 (0.997; 1.001)	0.999 (0.998; 1.000)		

OR = odds ratio; CI = confidence interval; MVPA = moderate-to-vigorous physical activity

^1^ number of zero observations: 218;

^2^ number of zero observations: 98;

^3^ number of zero observations: 258;

^4^ number of zero observations: 158;

^5^ number of zero observations: 239

Zero-inflated negative binomial models evaluate the correlates of the odds of non-participation in specific physical activity behaviors (logit model). Simultaneously, among the participants who performed the physical activity behavior, zero-inflated negative binomial models evaluate the correlates of weekly minutes of physical activity (negative binomial model). Negative binomial model parameters represent the proportional increase in minutes/week of the physical activity behavior with a one-unit increase in the predictor.

The negative binomial models (Table 3) showed that among those who cycled for transport during the last seven days, having a lower BMI, having no partner and having no college/university degree was associated with more cycling. Similarly, among those who walked for transport during the last seven days, having no college/university degree was associated with more walking. Furthermore, among those who cycled for recreation, male participants cycled more than female participants. Among those who walked for recreation during the last seven days, participants without a partner walked more than those with a partner. Finally, among those who participated in leisure-time MVPA, being male and having a lower BMI was associated with more leisure-time MVPA.

### Main associations of former work-related behaviors with sedentary behaviors and physical activity

The Gaussian models showed that higher levels of former work-related sitting were associated with more screen time during retirement. Work-related standing, walking and vigorous physical activity were not related to screen time during retirement.

None of the predictors significantly explained passive transportation during retirement.

For cycling for transport, the logit model yielded no significant results. The negative binomial model showed that among those who cycled for transport during the last seven days, lower levels of former work-related vigorous physical activity were associated with more cycling for transport. None of the other work-related behaviors were significant predictors of min/week of cycling for transport. For walking for transport, the logit model showed that participants with higher levels of former work-related walking were more likely to do any walking for transport during retirement. The negative binomial model showed that among those who walked for transport during the last seven days, higher levels of former work-related walking were predictive of higher levels of walking for transport during retirement. None of the other predictors in the logit and negative binomial models, were significantly associated with walking for transport. For cycling for recreation, walking for recreation and leisure-time MVPA no significant associations were found.

### Moderating effects of educational level and physical functioning

The significant moderating effects of educational level and physical functioning on the associations of former work-related behaviors with sedentary time and physical activity during retirement are presented in Table 4. Former work-related sitting was only positively associated with screen time in adults without physical limitations. Three moderating effects were found for the relationship with passive transport, but separate regression models in the subgroups did not show significant associations.

**Table 4:** Significant moderating effects of educational level and physical functioning on the associations of former work-related activity levels with sedentary time and physical activity during retirement.

Dependent variable	Model	Interaction(s)	β / OR (90% CI)^1^	Regression model for separate groups	
				Groups	β / OR (95% CI)^1^
Screen time	Gaussian	Physical functioning * sitting work	(β) -25.93 (-50.37;-1.56)	Functionally limited	0.90 (-0.02; 1.82)
				**Not functionally limited**	**2.07 (1.03; 3.11)**
Passive transport	Gaussian	Education * standing work	(β) 17.92 (2.54; 33.30)	No college/university	-0.60 (-1.34; 0.14)
				College/university	0.06 (-0.50; 0.62)
	Gaussian	Education * vigorous PA work	(β) 13.64 (0.26; 27.02)	No college/university	-0.31 (-0.80; 0.18)
				College/university	0.38 (-0.48; 1.24)
	Gaussian	Physical functioning * vigorous PA work	(β) 18.85 (4.20; 33.50)	Functionally limited	-0.33 (-1.27; 0.61)
				Not functionally limited	0.16 (-0.27; 0.59)
Cycling transport	ZINB	Education * walking work	(β) 1.08 (1.04; 1.12)	**No college/university**	**0.998 (0.996; 0.999)**
				College/university	1.002 (0.999; 1.004)
	ZI logit	Physical functioning * standing work	(OR) 0.93 (0.86; 0.99)	**Functionally limited**	**0.997 (0.994; 0.999)**
				Not functionally limited	1.001 (0.998; 1.004)
Walking transport	ZINB	Physical functioning * standing work	(β) 0.96 (0.93; 0.99)	Functionally limited	0.999 (0.998; 1.001)
				**Not functionally limited**	**1.002 (1.001; 1.003)**
Cycling recreation	ZINB	Education * sitting work	(β) 0.91 (0.86; 0.96)	**No college/university**	**1.003 (1.001; 1.004)**
				**College/university**	**0.998 (0.996; 0.999)**
	ZINB	Education * standing work	(β) 1.06 (1.01; 1.11)	**No college/university**	**0.997 (0.996; 0.999)**
				College/university	1.001 (0.999; 1.003)
	ZINB	Education * vigorous PA work	(β) 1.07 (1.03; 1.11)	**No college/university**	**0.997 (0.996; 0.999)**
				College/university	1.001 (0.998; 1.004)
	ZINB	Physical functioning * vigorous PA work	(β) 0.95 (0.91; 0.99)	**Functionally limited**	**0.998 (0.997; 0.999)**
				Not functionally limited	1.001 (0.998; 1.004)
Walking recreation	ZI logit	Education * standing work	(OR) 0.90 (0.84; 0.96)	No college/university	1.003 (0.999; 1.005)
				**College/university**	**0.997; 0.995; 0.999)**
	ZINB	Education * walking work	(β) 1.05 (1.01; 1.09)	No college/university	1.000 (0.998; 1.002)
				**College/university**	**1.003 (1.001; 1.005)**
	ZINB	Education * vigorous PA work	(β) 1.04 (1.01; 1.07)	No college/university	0.999 (0.998; 1.001)
				College/university	1.002 (0.999; 1.004)
Leisure-time MVPA	ZINB	Education * walking work	(β) 1.05 (1.01; 1.10)	No college/university	0.998 (0.995; 1.001)
				**College/university**	**1.003 (1.001; 1.005)**
	ZINB	Physical functioning * vigorous PA work	(β) 1.06 (1.02; 1.11)	Functionally limited	0.999 (0.998; 1.001)
				**Not functionally limited**	0.998 (0.996; 1.001)

ZINB: zero-inflated negative binomial model (count model); ZI logit: zero-inflated logit model (zero model); OR: odds ratio

^1^ β-coefficients are reported for results of the Gaussian and zero-inflated negative binomial models (count models), odds ratio’s are reported for results of the zero-inflated logit models (zero models)

*Note*: 90% confidence intervals are used to determine significance of interaction effects because these analyses have lower statistical power [26]

Zero-inflated negative binomial models evaluate the correlates of the odds of non-participation in specific sedentary/physical activity behaviors (logit model). Simultaneously, among the participants who performed the sedentary/physical activity behavior, zero-inflated negative binomial models evaluate the correlates of weekly minutes of sedentary time/physical activity (negative binomial model). Negative binomial model parameters represent the proportional increase in minutes/week of the sedentary/physical activity behavior with a one-unit increase in the predictor.

Regarding cycling for transport, two moderating effects were found: among the adults who cycled for transport (negative binomial model), work-related walking was only negatively related to cycling for transport in those without a college/university degree. Furthermore the logit model showed that adults with higher levels of former work-related standing were more likely to cycle for transport, but this association was only significant in adults who were physically limited. Regarding walking for transport, the negative binomial model showed that among adults who walked for transport, more work-related standing was related to more walking for transport, but only in adults without physical limitations.

The negative binomial models with cycling for recreation as the dependent variable revealed four moderating effects. Among the participants who cycled for recreation during the last seven days, work-related standing and work-related vigorous physical activity were only negatively related to cycling for recreation in adults without a college/university degree. Furthermore, higher levels of work-related sitting were associated with more recreational cycling in adults without a college/university degree, but with less recreational cycling in adults with a college/university degree. Finally, former work-related vigorous physical activity was only negatively related to cycling for recreation in adults with physical limitations.

Regarding walking for recreation, the logit model showed that only in adults with a college/university degree, those with higher levels of former work-related standing were more likely to walk for recreation. Furthermore, the negative binomial model showed that among adults who walked for recreation, work-related walking was positively related to walking for recreation, but only in adults with a college/university degree.

Finally, regarding leisure-time MVPA, the negative binomial model showed that among those who did leisure-time MVPA, more work-related walking was associated with more leisure-time MVPA but only in adults with a college/university degree.

## Discussion

To our knowledge, this was the first study examining the current research questions, making it difficult to compare the present results to previous study findings. The first study aim was to examine if former work-related behaviors (sitting, standing, walking and vigorous activities) predicted physical activity and sedentary time during early retirement. Overall, very few significant associations were revealed, indicating that the role of former work-related behaviors in determining physical activity and sedentary time during early retirement is limited.

When examining the results in detail, a first notable finding is the absence of associations between former work-related activities and leisure-time physical activity (walking, cycling and MVPA) during retirement. This finding is not in line with the hypothesis that was previously formulated by Barnett and colleagues [15], stating that adults who had a physically demanding job attach low personal value to leisure-time physical activity and experience retirement as a period of well-deserved rest. On the contrary, our findings showed that former work-related physical activity and sitting time do not affect leisure-time physical activity during retirement; probably other factors (e.g. former total physical activity, visions on ageing, perceived benefits and barriers towards physical activity, social support, social networks, living environment) [15,16,19] are more important.

Secondly, for active transport during retirement, the results were somewhat more in line with the expectations and previously formulated hypotheses although again, only a limited number of significant associations were identified. Individuals with high levels of former work-related vigorous physical activity spent less time on cycling for transport during retirement, while those who performed light-to-moderate physical activity (i.e. walking) at work, seemed to continue this healthy habit with higher levels (both higher odds and more min/week) of walking for transport during retirement. Based on these findings, it seems that the mechanisms explaining active transportation during retirement differ from those explaining leisure-time physical activity; this may be due to the fact that leisure-time physical activity is more governed by choice than by necessity or habit [29], which possibly implies that mainly psychosocial factors are important to explain leisure-time physical activity during retirement.

A third notable finding was that higher levels of work-related sitting were predictive of more screen time during retirement, so it seems that the unhealthy habit of being sedentary at work is continued during retirement, with high levels of screen time. This is important for future interventions: mainly individuals with a sedentary job should be made aware of the importance and the positive health consequences of limiting sedentary time during retirement.

The second aim of this study was to examine the moderating effects of educational level and physical functioning on the previously discussed associations. Both for educational level and physical functioning, interactions with former work-related behaviors were found, but the direction of the interactions was not univocal. The moderators mainly played a role to explain physical activity during retirement (11 significant interactions), and not to explain sedentary time during retirement (only one significant interaction).

The moderating effects of educational level partly confirm the hypothesis that lower-educated individuals with a physically demanding job are susceptible to lapsing into an inactive lifestyle after retirement [9,30]: more work-related standing, walking and vigorous physical activity were predictive of less cycling (for transport or recreation) during retirement. However, higher levels of former work-related sitting were associated with more cycling for recreation during retirement in lower-educated individuals, which contradicts the previous findings. In higher-educated individuals, results are also conflicting: some of the former work-related activities were associated with more, and others with less physical activity during retirement. Positive effects of former work-related walking on leisure-time walking and MVPA during retirement in higher-educated adults were found; this might be due to the fact that these individuals are more conscious of the health benefits of physical activity and try to continue the healthy working habits during retirement. Furthermore, higher-educated individuals probably have more financial and organizational opportunities (e.g. availability of a car) that make it possible to participate in organized leisure-time activities like walking tours or sports activities. Nonetheless, nor for the higher-educated adults clear univocal results were available, making it impossible to give clear recommendations for future interventions (e.g. whether or not to focus on specific subgroups).

Similarly, no consistent results were found for the moderating effects of physical functioning. Both in functionally limited and unlimited adults, more work-related standing was predictive of more active transport during retirement, while more work-related vigorous activity was associated with less leisure-time physical activity during retirement. A possible explanation for these findings could be that overall, the physical health of recently retired adults aged between 58 and 65, is rather good. Other results may emerge if a similar study would be conducted in older adults (+65 years). Nonetheless, like for educational level, the contradictory moderating effects found for physical functioning make it impossible to formulate concrete recommendations for future interventions in adults making the transition to retirement.

A first strength is that this was the first study to include former work-related activity levels as predictors of physical activity and sedentary time during retirement. Previous studies suggested that work-related physical activity could be an important predictor, but only used employment category as a proxy of occupational physical activity to verify this hypothesis [9,17]. Furthermore the statistical approach that was used (zero-inflated regression models) takes into account the large number of zero values reported for the physical activity variables, which is often not done in other studies but is necessary to obtain reliable results. However, an inherent problem of this approach is that results become more complex, with specific determinants for specific outcomes (no versus any activity and duration among those who perform the activity), making the formulation of concrete recommendations for future interventions more challenging. Furthermore, some limitations should be acknowledged. First, former work-related behaviors were assessed retrospectively, which may induce recall bias and can lead to misreporting. Second, only former work-related physical activity was assessed, and not overall physical activity before retirement. Overall physical activity before retirement may be a stronger predictor of physical activity during retirement and should be included in future studies, although it will be even more challenging to retrospectively assess this behavior accurately. Third, retirement is a complex concept, and is defined differently across studies (e.g. only inclusion of individuals who are not involved in voluntary work, or also including who still do part-time work after retirement), making comparison of results across studies difficult [17]. Fourth, the absence of significant associations may be partly due to a lack of statistical power, as post-hoc power analyses showed that for three of the nine regression models (MVPA, cycling for transport and passive transportation), statistical power was somewhat lower than 0.80. In future studies, a larger study sample would be needed to overcome this problem and to draw firm conclusions. Fifth, unassessed psychological confounders possibly have an important impact on our results, so it would be interesting to assess such variables in future studies. Finally, selection bias was probably present as only 428 adults out of the 7500 who received an information letter, completed the questionnaire and as the sample was more-highly educated than the general Belgian population. These issues may limit the generalizability of our findings.

## Conclusions

In conclusion, the current study showed that former work-related behaviors are not very important to explain physical activity during retirement, so future studies should further examine the importance of other individual, social and environmental determinants, as well as former leisure-time or overall physical activity in order to be able to develop effective interventions to prevent retired adults from lapsing into an inactive lifestyle. Only for screen time during retirement, we found that adults who previously had a sedentary job are more susceptible to maintaining this sedentary lifestyle during retirement, so this is an important subgroup to focus on during interventions. Some moderating effects of educational level and physical functioning were found, but since the direction of the interactions was not univocal, no clear recommendations could be formulated. Future research in a more representative sample of retired adults may further clarify these moderating effects.

## Supporting Information

S1 Dataset Supporting information for article. (SAV)
